# Analysis of pollen-specific alternative splicing in *Arabidopsis thaliana* via semi-quantitative PCR

**DOI:** 10.7717/peerj.919

**Published:** 2015-04-28

**Authors:** April D. Estrada, Nowlan H. Freese, Ivory C. Blakley, Ann E. Loraine

**Affiliations:** Department of Bioinformatics and Genomics, North Carolina Research Campus, University of North Carolina at Charlotte, Charlotte, NC, USA

**Keywords:** Pollen, Alternative splicing, Arabidopsis, SR protein, RNA helicase, Bioinformatics, RNA-Seq, Integrated Genome Browser

## Abstract

Alternative splicing enables a single gene to produce multiple mRNA isoforms by varying splice site selection. In animals, alternative splicing of mRNA isoforms between cell types is widespread and supports cellular differentiation. In plants, at least 20% of multi-exon genes are alternatively spliced, but the extent and significance of tissue-specific splicing is less well understood, partly because it is difficult to isolate cells of a single type. Pollen is a useful model system to study tissue-specific splicing in higher plants because pollen grains contain only two cell types and can be collected in large amounts without damaging cells. Previously, we identified pollen-specific splicing patterns by comparing RNA-Seq data from Arabidopsis pollen and leaves. Here, we used semi-quantitative PCR to validate pollen-specific splicing patterns among genes where RNA-Seq data analysis indicated splicing was most different between pollen and leaves. PCR testing confirmed eight of nine alternative splicing patterns, and results from the ninth were inconclusive. In four genes, alternative transcriptional start sites coincided with alternative splicing. This study highlights the value of the low-cost PCR assay as a method of validating RNA-Seq results.

## Introduction

Alternative splicing is a form of post- and co-transcriptional regulation in which pre-mRNA transcripts from the same gene are spliced in different ways. Alternative splicing can affect any part of a transcript, and it has been well known since the early 2000s that alternative splicing in mammalian genomes frequently remodels conserved or functional motifs in the encoded proteins (Loraine et al., 2002; Cline et al., 2004). These differences can have important functional consequences; in animals as diverse as fruit flies and mammals, differential expression of alternative splice variants supports and enforces cellular differentiation and development. Textbook examples of regulatory processes that depend on differential expression of splice variants include sex determination in insects, neuronal differentiation in mammals, and aspects of hormone signaling (reviewed in Li, Lee & Black, 2007; Zheng & Black, 2013).

In higher plants, far less is known about tissue or cell-type specific alternative splicing. This is partly due to a lack of data, as well as the technical difficulty in collecting large amounts of the same cell type from plants. Until recently, ESTs were the main source of information about splice variants, and several studies used ESTs to investigate the diversity and prevalence of alternative splicing in plants (Ewing et al., 1999; Campbell et al., 2006; Schindler et al., 2008; Wang et al., 2008b; English, Patel & Loraine, 2010). However, in contrast to human and mouse genomes, for which tens of millions of ESTs are available, the best-studied plants (rice, maize, and Arabidopsis) have fewer than four million ESTs combined, which meant that far less has been learned about alternative splicing in plants compared to animals. Now, thanks to high throughput cDNA sequencing (RNA-Seq) and improving bioinformatics approaches, limited data is becoming less of a problem. However, another challenge arises from the unique biology of plants in comparison to animals; plant cells inhabit a complex matrix of cell wall materials that make it difficult to prepare homogenous cell populations. The integrity and composition of the cell wall plays a key role in regulation of plant cellular processes, especially defense responses, which involve changes in splicing patterns (Howard et al., 2013). Thus, there is a possibility that cell isolation methods that involve degradation of the cell wall may introduce artifacts leading to over- or under-estimation of tissue- or cell-type specific alternative splicing.

Fortunately, there are some tissue or cell types in plants where the sample collection problem can be overcome. Pollen grains are one example; in most plants, pollen is well suited to collecting cellular materials in amounts required for preparing cDNA libraries for RNA-Seq (Johnson-Brousseau & McCormick, 2004). During pollen development, the pollen grain becomes dehydrated and enters a dormant state, and so mature pollen can be collected in large amounts with minimal risk of introducing artifacts, in part because collection procedures are typically no harsher than natural mechanisms by which pollen is spread by insects or wind. In Arabidopsis, similar to other flowering plants, pollen grains contain three cells: two sperm cells and a vegetative cell, also called the pollen tube cell, which encloses the sperm cells and gives rise to the pollen tube (reviewed in Dresselhaus & Franklin-Tong, 2013). When a pollen grain lands on a compatible stigma, the grain rehydrates and germinates, extruding a pollen tube that grows through the style toward the ovary, carrying the sperm cells. Upon entering the ovary, the pollen tube bursts, releasing the sperm, which merge with the central cell and the egg, giving rise to endosperm and embryo. Experiments with *in vitro* pollen germination and inhibitors of transcription have shown that Arabidopsis pollen grains contain sufficient mRNA to support the early stages of germination and tube growth, but synthesis of new RNA is required to complete fertilization (Honys & Twell, 2003; Wang et al., 2008b) with gene expression changing during tube growth (Honys & Twell, 2003; Honys & Twell, 2004; Pina et al., 2005; Wang et al., 2008b; Qin et al., 2009; Boavida et al., 2011). Thus pollen offers an opportunity to identify and study tissue-specific splicing in plants and how splicing changes during a well-studied developmental process.

Previously we used high throughput sequencing of cDNA (RNA-Seq) to investigate gene expression and splicing patterns in Arabidopsis pollen (Loraine et al., 2013). By comparing gene models and RNA-Seq read alignments, we identified around thirty genes that were annotated as alternatively spliced, were expressed in both pollen and leaves, and where the relative abundance of splice site use differed between pollen and leaves. Thus, these genes represented candidates for tissue-specific splicing in Arabidopsis. Here, we report results from new experiments that tested alternative splicing of a subset of these genes using semi-quantitative PCR. Our results confirmed tissue-specific splicing patterns, and also indicated that for many genes where alternative splicing affected the 5′ region, alternative transcriptional start sites (TSSs) may also play an important role in generating transcript diversity.

## Materials and Methods

### Bioinformatic analysis of splicing patterns

Our previously published report described using RNA-Seq alignments to identify differential alternative splicing in pollen and leaf RNA-Seq data sets (Loraine et al., 2013). Briefly, alternative splicing (AS) events annotated as part of the TAIR10 gene models from *Arabidopsis thaliana* were identified by comparing regions of overlap between exons and introns in different gene models belonging to the same gene, as described in English, Patel & Loraine (2010). For each alternative splicing event, the number of spliced reads supporting mutually exclusive choices was identified for each of two leaf libraries and one pollen library. For simplicity, only annotated alternative donor and acceptor sites were considered, and alternative splice forms were compared as pairs. For each AS event, the percentage of gapped reads in each library that supported each form was calculated and compared between pollen and leaf samples. Alternative splicing events were identified in which percentage of reads supporting a given splicing choice varied more than 20% between the two sample types. The AS events were then ordered by this percent difference; the listing of these events is shown in Data S1. The ten most differentially spliced genes were identified for testing by PCR; the list included At-U2AF65A, which was tested previously and is not discussed again here (Loraine et al., 2013). Note also that previously we referred to the leaf samples as “seedlings,” but here refer to them as “leaf” samples. This is because the older terminology was confusing to some readers. For many people the term “seedling” means: whole seedlings that are two weeks old or younger. The samples used in here and in the previous study were from three-week old, soil-grown plants and included leaves and not roots.

### PCR primer design for analysis of splicing patterns

Primers flanking the differentially spliced region were designed using the Primer3 software deployed on the NCBI Web site. Primer locations within the target gene were visualized using Integrated Genome Browser (Nicol et al., 2009) and amplicon sizes were calculated for each splice variant. Where possible, primers were designed to have predicted melting temperatures of 60 °C.

### Pollen collection

Pollen RNA samples were collected and prepared as described in Johnson-Brousseau & McCormick (2004) and Loraine et al. (2013). Pollen samples corresponding to samples P1, P2, and P3 were collected by vacuuming flowers from different sets of pots growing under 16 h/8 h light/dark illumination. P1 and P2 were collected in 2012 and P3 was collected in 2008. P3 was also the same sample used to synthesize pollen cDNA that was used for sequencing (Loraine et al., 2013). Pollen samples were frozen following collection and stored at −80 °C prior to use.

### Arabidopsis growth conditions

Leaf samples used in this study corresponded to non-stressed controls from pot “L” described in Gulledge et al. (2012). The above-ground parts of plants (leaves but not roots or stems) of plants growing on soil were collected after 21 days of growth under 16/8 light/dark illumination in an incubator (model AR36L3 from Percival Scientific, Perry, Iowa, USA) set to 22 °C, 45% relative humidity, and 150 photosynthetic photon flux density (PPFD). The plants had not yet bolted at the time of collection.

### RNA extraction

Frozen pollen or leaf samples were ground in liquid nitrogen with a mortar and pestle and RNA was extracted using a RNeasy kit (Bio-Rad catalog number 74904: Bio-Rad; Hercules, California, USA) following manufacturer’s instructions. RNAs were DNaseI treated via an on-column digestion with the Plant RNeasy Kit. RNA quality was assessed with an Agilent BioAnalyzer and only high quality samples were used.

### cDNA synthesis

cDNA samples were synthesized following the SMART MMLV reverse transcriptase kit protocol (Company is Clonetech, catalog number 639537; Clontech, Mountain View, California, USA), using the supplied 5X buffer and DTT supplied with the kit, 50 mM oligo dT primers (Bioline, catalog number BIO-38029; Bioline, Taunton, Massachusetts, USA) and 10 mM dNTPs (Bioline, cat. no. 39053; Bioline, Taunton, Massachusetts, USA). 1 µg of total RNA was used in 20 µL cDNA synthesis reactions.

### PCR amplification of cDNAs from pollen and leaf cDNAs

PCR amplifications included an initial “hot start” 4 min denaturation incubation at 94 °C followed by 30 cycles of denaturation (94 °C), annealing, and elongation (72 °C) incubations. Annealing temperatures were selected based on Tm predicted by the Primer 3 software, and when primers failed to amplify a band, gradient PCR was performed to identify the optimal annealing temperature. Amplifications were typically performed in volumes of 20 µL per template/primer combination with 2 µL each of 5 µM forward and reverse primer stock solutions, .08 µL of taq polymerase (Bioline, catalog number 21086; Bioline, Taunton, Massachusetts, USA), and 0.5 µL aliquots of pollen or leaf cDNA reactions that had been diluted 1:20. Cycle numbers were estimated to be in logarithmic phase based on prior analysis of qPCR analysis of genes of varying starting RPKMs (Loraine et al., 2013).

### Analysis of gel images for semi-quantitative PCR

The relative abundance of splice variants amplified by PCR was estimated using methods presented in Palusa, Ali & Reddy (2007) and Venables et al. (2012). Equal amounts of PCR reaction mixtures were fractionated by agarose gel electrophoresis containing ethidium bromide. Gels were visualized under short wave UV light in a UVP Biospectrum AC Imaging System GelDoc station and photographed using a digital camera. Each gel was typically photographed at different run times and using different exposure times to obtain high quality images for analysis.

Gel images were analyzed using the VisionWorksLS version 7.0.1 software program. Rectangular regions surrounding ethidium-stained bands in each lane were identified, and for each band, the software was used to estimate molecular weight along with a background-corrected band intensity value, called “I-vol” in the software. Visualization of lane profiles was also done to ensure that band intensity measurements were within the range of detection of the instrument. Gels also included lanes containing molecular mass standards (Hyperladder 1 kb, Bioline catalog number BIO-33053; Bioline, Taunton, Massachusetts, USA). Band intensities of the molecular size/mass markers were plotted against their masses and only gels where this relationship was close to linear (*R*^2^ > = 0.90) were used. Band intensities and size estimates were then imported into the R statistical programming environment for analysis.

For each band, its abundance relative to other bands in the same lane was calculated and used to test for differences between pollen and leaf samples. The relative abundance of per band was calculated as its percent contribution the total amount of DNA in each lane. The percent contribution per band was calculated as 100 × *b*/*T*, where *b* was the band’s size-normalized intensity and *T* was the sum of all size-normalized band intensities in the lane, calculated as $\sum _{i=1}^{N}{b}_{i}$. Size-based normalization of band intensity was done to account for how ethidium staining (and band brightness) depended on both amplicon abundance and amplicon size, since larger amplicons contained more sites (base pairs) for ethidium to bind (Green & Sambrook, 2012). For each gel containing replicate pollen and leaf samples, a *t* test was used to determine whether there was a statistically significant difference in a band’s percentage between pollen and leaf samples. As the prior RNA-Seq data analysis indicated whether pollen or leaf percentages were greater and since there was no reason to expect unequal variance, one-sided *t*-tests assuming equal variance were used. R code used to calculate percentages and perform statistical testing are available as Markdown (.Rmd) in the folder named “GelAnalysis” in the project repository at http://www.bitbucket.org/lorainelab/pollenas. For each gene tested, a Markdown file and its corresponding HTML report file is available; readers can re-run the code or open the HTML file in a Web browser to view the code, the output of the code, and accompanying figures.

### Sanger sequencing of PCR products

For higher-abundance amplicons that did not match annotated gene models or that suggested use of alternative transcription start sites, PCR products were extracted from the gels and sequenced by conventional Sanger sequencing to confirm band identity. Sequence reads obtained from Sanger sequencing were aligned on the Arabidopsis genome using blat (Kent, 2002) and visualized in Integrated Genome Browser for comparison with gene models. Blat was run with default settings except for the maxIntron parameter, which was set to 2,000, since 99% of Arabidopsis introns are 2,000 bases or smaller. Sequencing results are described in detail in the folder named “AmpliconSequencing” in the project repository at http://www.bitbucket.org/lorainelab/pollenas.

### Data access

The RNA-Seq data are available from the Sequence Read Archive under SRA accessions SRR847501, SRR847502 (pollen library); SRR847503, SRR847504 (leaf library 1); and SRR847505, SRR847506 (leaf library 2). Read and junction feature alignments depicted in figures are available from the IGBQuickLoad data source named “pollen” under the Arabidopsis June 2009 (TAIR9/10) genome assembly release. A tutorial introducing Integrated Genome Browser and describing how to perform a visual splicing analysis using the pollen RNA-Seq data set is available at http://tinyurl.com/pk25nbm.

## Results

To further investigate pollen-specific splicing patterns, we first revisited the analysis described in our previous study of gene expression in pollen using RNA-Seq data (Loraine et al., 2013). We identified 30 genes (Data S1) that were expressed in both pollen and leaves where the prevalence of at least one splicing variant differed 20% or more between the two samples types. From this list, we selected the most highly differentially spliced genes for further testing via semi-quantitative PCR, an established method for assaying alternative splicing (Ali et al., 2007; Venables et al., 2012). In semi-quantitative PCR, primers that flank the differentially spliced region are used to amplify splice variants from cDNA prepared from different samples, ideally multiple biological replicates. PCR products are run out on a gel, visualized by staining with ethidium bromide, and then the relative brightness of each amplicon (band) is quantified using image analysis software (Green & Sambrook, 2012). The percent contribution of each band to the total signal in each lane is compared across lanes; if the percent abundance of a band differs between samples, e.g., 10% vs. 90% in pollen and leaf, this indicates difference in splicing or in some other aspects of transcript structure.

An advantage of semi-quantitative PCR for assessing splicing changes is that because the values being compared are percentages, expressed relative to the total amount of product per lane, the amount of cDNA used as template in each reaction can vary between lanes; no across-lane normalization is required. Another advantage is that PCR and gel electrophoresis are inexpensive and do not require specialized equipment. Using this method, we previously confirmed that one of the ten most differentially spliced genes (U2AF65A) was differentially spliced between pollen versus leaves. Here, we used semi-quantitative PCR supplemented with Sanger sequencing of amplicons to test differential splicing of the nine other most differently spliced genes identified by RNA-Seq.

### Splice variants from Arabidopsis SR genes At-SR30 (AT1G09140) and At-RS41 (AT5G52040) are differentially spliced in pollen and leaves

**Figure 1:**
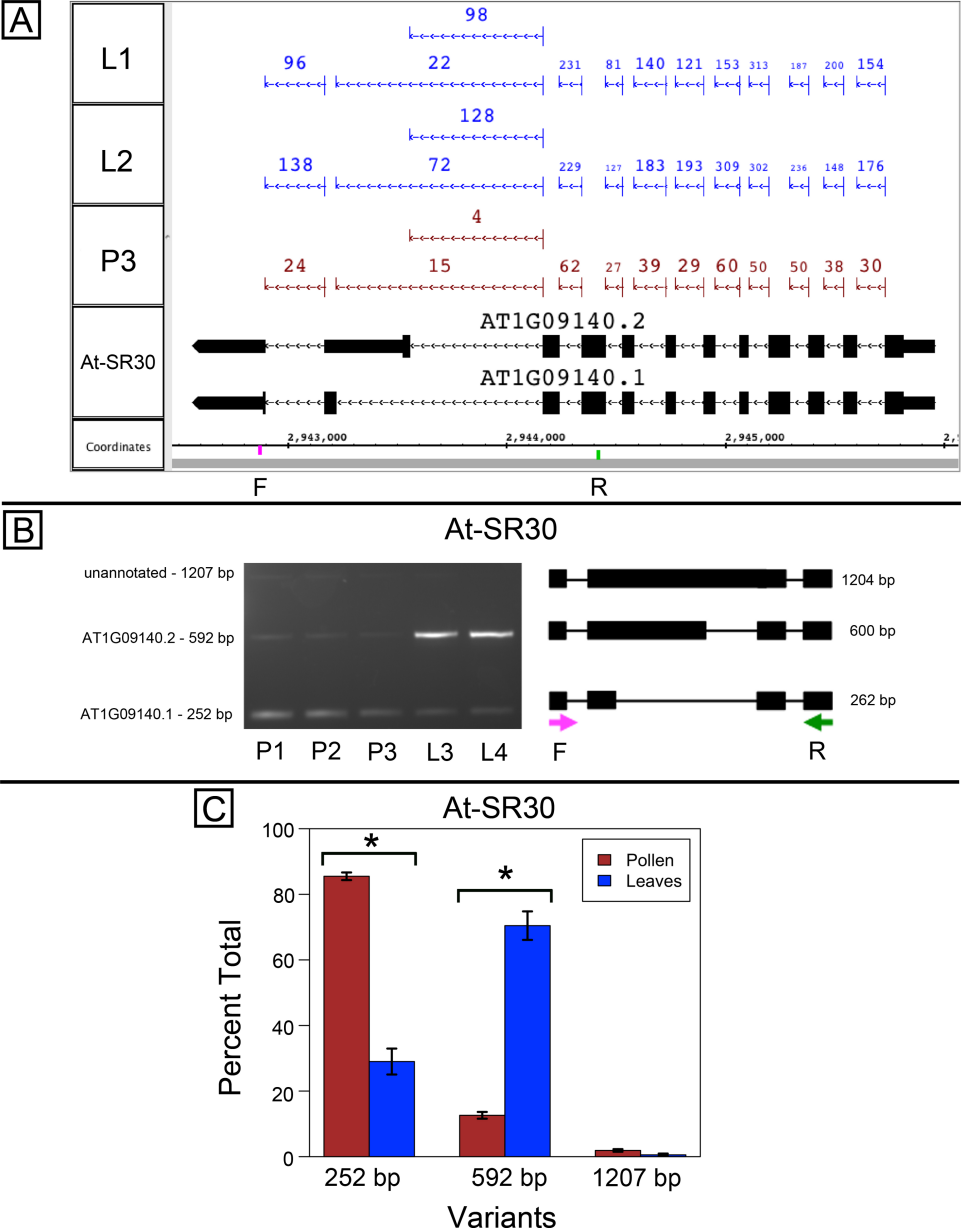
Pollen-specific splicing in At-SR30. (A) Junction features from RNA-Seq reads for leaf (L1, L2) and pollen (P3) samples alongside annotated gene models. Numbers indicate how many spliced reads supported the indicated junction in pollen and leaf RNA-Seq libraries. Arrows indicate the direction of transcription and taller blocks show translated regions. Primers used in semi-quantitative PCR are shown. Primer sequences were CCAGTGGCCAGTTTTCATTT (F) and GTGTGAGTCGAAGCCCAGAT (R). (B) Gel electrophoresis of PCR amplification of pollen (P1, P2, P3) and leaf (L3, L4) cDNAs and corresponding model of alternative splice variants. Estimated fragment size from gel and theoretical fragment size based on splice model found to left and right, respectively, in base pairs (bp). (C) Percent total of each observed splice variant in pollen and leaf samples quantified from gel electrophoresis in (B). Values are averages of replicate samples. Error bars indicate two standard deviations. Asterisk indicates *p*-value less than 0.05, double asterisk less than 0.01.

**Figure 2:**
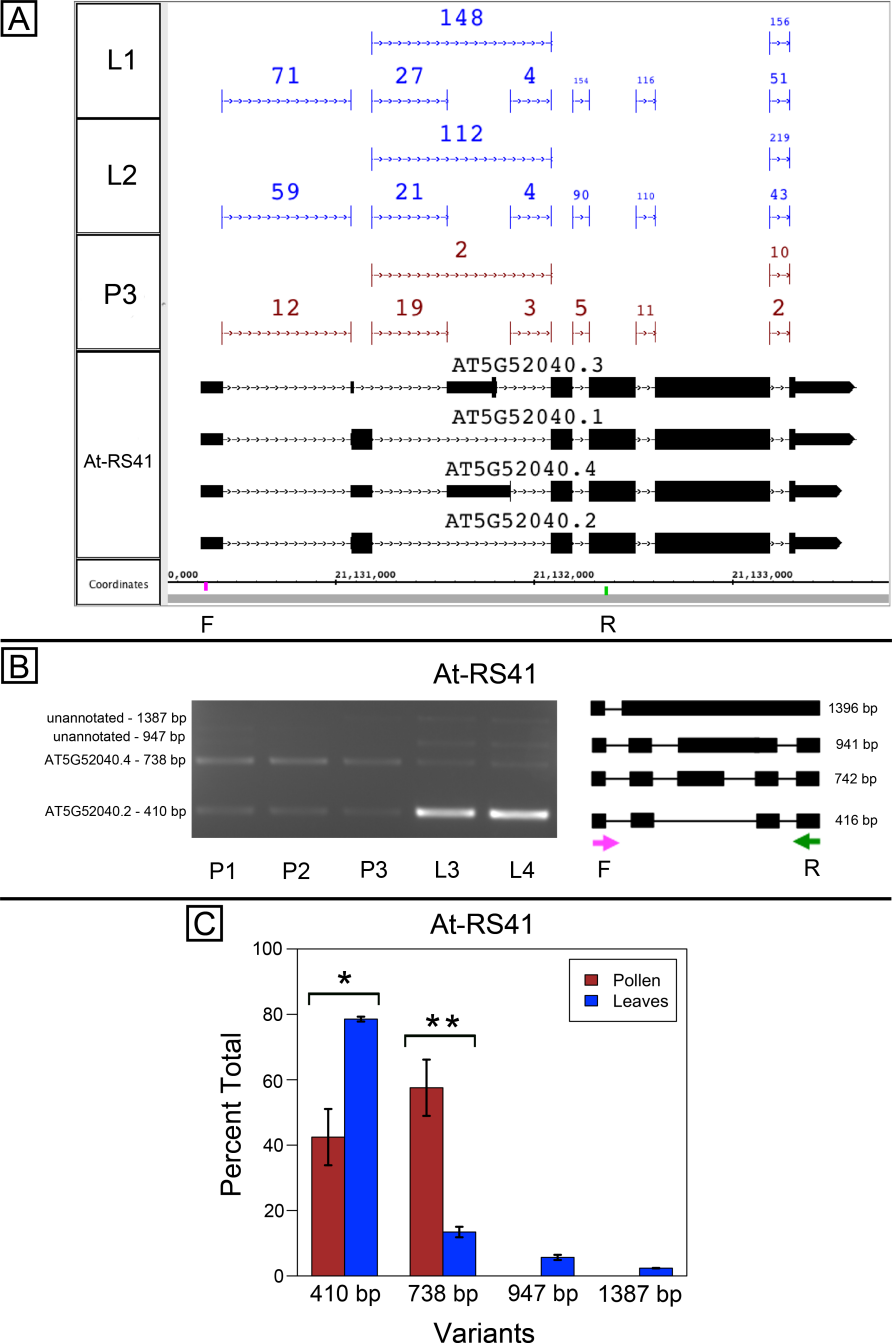
Pollen-specific splicing in At-RS41. (A) Junction features from RNA-Seq reads for leaf (L1, L2) and pollen (P3) samples alongside annotated gene models. Numbers indicate how many spliced reads supported the indicated junction in pollen and leaf RNA-Seq libraries. Primers used in semi-quantitative PCR are shown. Primer sequences were GAGAGCCTCGAAGAAAGCAA (F) and TGCATCGAAGTTGATCACAA (R). (B) Gel electrophoresis of PCR amplification of pollen (P1, P2, P3) and leaf (L3, L4) cDNAs and corresponding model of alternative splice variants. Estimated fragment size from gel and theoretical fragment size based on splice model found to left and right, respectively, in base pairs (bp). (C) Percent total of each observed splice variant in pollen and leaf samples quantified from gel electrophoresis in (B). Values are averages of replicate samples. Error bars indicate two standard deviations. Asterisk indicates *p*-value less than 0.05, double asterisk less than 0.01.

At-SR30 and At-RS41 encode two of eighteen serine/arginine-rich (SR) RNA-binding proteins in Arabidopsis (Barta, Kalyna & Reddy, 2010) thought to play a role in regulation of splice site choice. According to the RNA-Seq data, At-SR30 (Fig. 1A) and At-RS41 (Fig. 2A) were differentially spliced in pollen and leaf. Figures 1A and 2A show junction features aligned onto each gene, where features summarize the number of spliced reads in each sample that supported the corresponding intron in the gene models. In both genes, the relative abundance of spliced reads supporting alternative splicing events was clearly different in leaf versus pollen. We also observed a small number of unspliced reads that aligned to the differentially spliced intron, indicating a low level of intron retention (not shown), a common form of alternative splicing in plants (English, Patel & Loraine, 2010).

To validate the RNA-Seq results, we designed PCR primers flanking the differentially spliced region and performed semi-quantitative PCR using newly prepared pollen and leaf cDNA samples. For both genes, semi-quantitative PCR confirmed the differential splicing patterns found by RNA-Seq. Figures 1B–1C shows that two splice variants of At-SR30 were expressed in both pollen and leaf samples, but the relative abundance of the two variants was different. We also observed a low abundance, higher molecular weight amplicon in pollen and leaf samples consistent with intron retention. For At-RS41, we observed similar results; PCR amplified four splice variants, the relative abundance of the splice variants was different between pollen and leaves, and low abundance amplicons consistent with intron retention were apparent (Figs. 2B–2C).

In both genes, differential splicing affected the protein-coding region of the transcripts, but in different ways. In At-SR30, the leaf-preferred isoform introduced a premature stop codon that deleted 23 amino acids from the carboxyl end of the protein, including five SR repeats, which are possible phosphorylation targets and mediate interactions with other proteins (reviewed in Shepard & Hertel, 2009). For At-RS41, the pollen-preferred isoform included an alternative exon that introduced a new translation start site leading to loss of 35 N-terminal amino acids encoding part of an RNA recognition motif. Thus, the pollen-preferred form of At-SR30 encoded the full-length protein, while the pollen-preferred form of At-RS41 encoded a truncated form lacking part of the amino terminal region of the protein.

### Alternative splicing in pollen remodels starch synthase gene AT5G65685

AT5G65685, encoding a starch synthase-like protein, is highly alternatively spliced in the 5′ region (Fig. S1A). Visualization of RNA-Seq alignments suggested there were many possible splice variants affecting the amino terminus of the protein and that the splicing patterns in pollen and leaves were different. According to the RNA-Seq data, the first intron (relative to isoform AT5G65685.1) contained two alternative donor sites that were differentially spliced in pollen compared to leaves, with leaf samples using the upstream site and pollen samples using the downstream site. The RNA-Seq data also suggested there were several unannotated variants containing retained introns. Results from semi-quantitative PCR were consistent with these observations (Figs. S1B–S1C). PCR primers designed around the alternatively spliced region amplified multiple bands corresponding to annotated variants and to many novel, lower abundance variants corresponding to retained intron isoforms.

### Alternative splicing of AT4G21720, gene of unknown function

AT4G21720 encodes a conserved protein of unknown function with two annotated splice variants, with alternative acceptor sites affecting the 3′ end of the second intron (Fig. S2A). Visualization of RNA-Seq read alignments in Integrated Genome Browser indicated that the upstream acceptor site, which introduced a frameshift that eliminated 44 amino acids, was preferred in pollen, and the downstream acceptor site, which allowed translation of the full-length protein, was preferred in leaves.

As before, primers flanking the alternatively spliced region were designed and used to amplify cDNAs prepared from pollen and leaves (Figs. S2B–S2C). The PCR assay results supported differential splicing of AT4G21720 in pollen versus leaves. PCR products consistent with both acceptor sites were observed and the relative abundance of the products was similar to what was observed in the RNA-Seq data (Figs. S2B–S2C and Data S1). An additional higher molecular weight band was also observed in both samples, and its size suggested it was derived from occasional retention of the alternatively splice intron. However, its abundance was extremely low.

### 5′ alternative splicing coincided with alternative TSSs in four of nine genes tested

**Figure 3:**
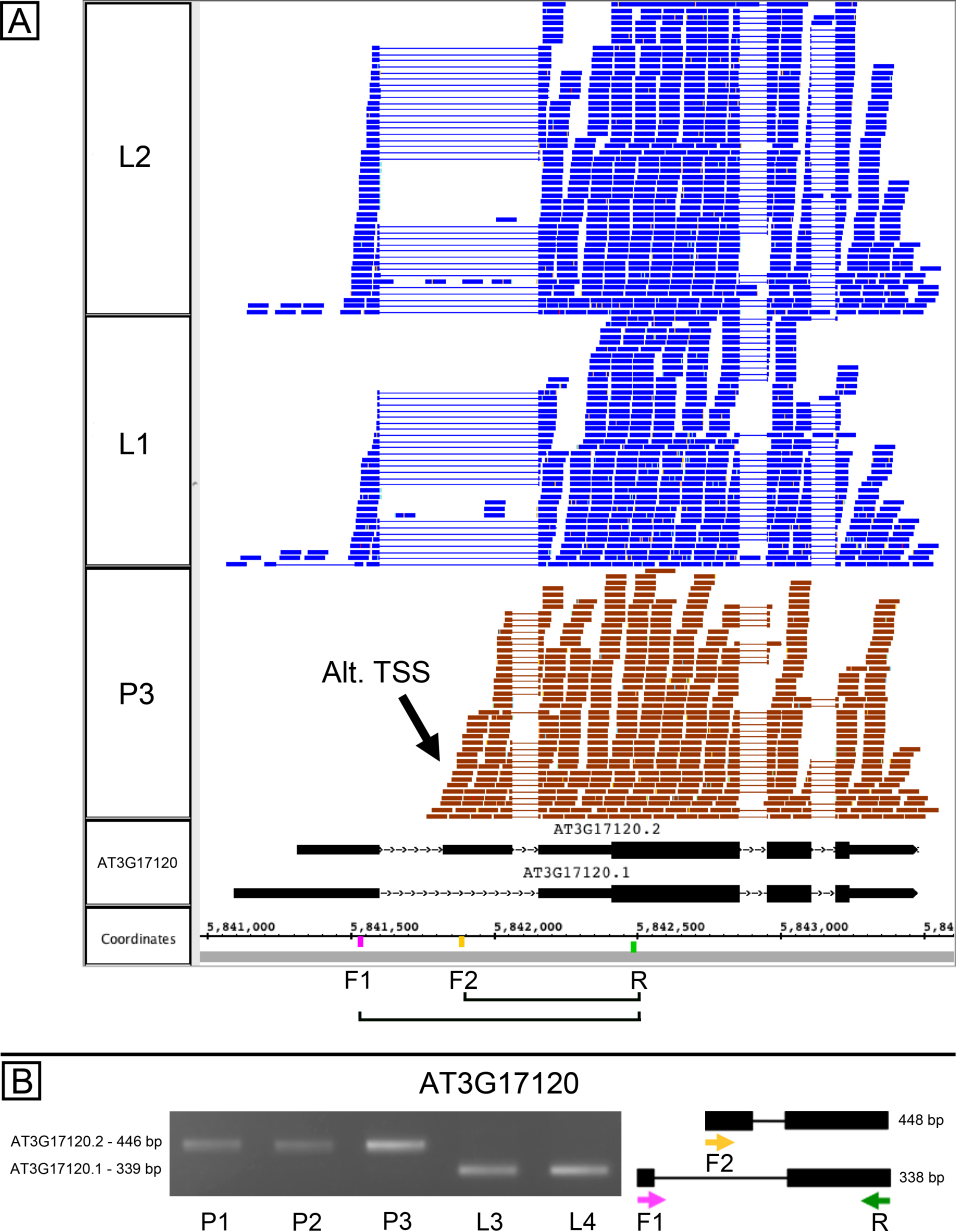
Alternative splicing and putative alternative transcriptional start site in AT3G17120, a gene of unknown function. (A) Visualization of RNA-Seq read alignments from leaf (L2, L1) and pollen (P3). Locations of PCR primers are indicated on the coordinates track. Primer sequences from left (5′) to right (3′) were AGAATCCGCCATTTTCTCCT (F1), CCTTGCTTCAAGCCCGAGAT (F2), and TTCCCATTATCTCCCCAGATT (R). (B) Gel electrophoresis of PCR products from amplifying pollen (P1, P2, and P3) and leaf (L1, L2) cDNAs in reactions that included primers F1, F2, and R. Corresponding model of alternative splice variants to right of gel. Estimated fragment size from gel and theoretical fragment size based on splice model found to left and right, respectively, in base pairs (bp). Asterisk indicates *p*-value less than 0.05, double asterisk less than 0.01.

AT3G17120, another gene of unknown function, is annotated as producing two splice variants due to exon skipping in the 5′ UTR of the gene, and the RNA-Seq data analysis indicated that the relative abundance of splice variants was different in pollen and leaves (Data S1). However, visual inspection of RNA-Seq alignments alongside the gene models suggested the gene contained an alternative transcription start site (TSS) located within the first annotated intron (Fig. 3A). We tested this in PCR experiments using variant specific primers F1 and F2 and a common primer R. In leaves, F1 with R amplified a product, but F2 and R did not (Fig. 3B). In pollen, F2 and R amplified a product, but F1 with R did not. Combining all three primers in the same reaction amplified leaf and pollen-specific products, and sequencing confirmed the identity of products shown in Fig. 3B. This confirmed that pollen and leaves expressed distinct isoforms and strongly suggested that the alternative transcripts arose from alternative transcriptional start sites. Because the suggested pollen alternative TSS was downstream of the 5′ end of the first intron, transcripts arising from this alternative TSS would therefore require a different 5′ donor site to be used in splicing. In this case, alternative transcription and alternative splicing were linked.

Alternative splicing of AT3G06330, encoding a putative RING-type ubiquitin ligase, also appeared to coincide with alternative TSSs. AT3G06330 is annotated as having an alternative donor site in the 5′ end of the gene that changes the amino terminal region of the protein. According to the RNA-Seq data, the downstream donor site was preferred in pollen, while the upstream donor site was preferred in leaves (Fig. S3A). As before, we designed variant-specific forward primers and used them together with a common reverse primer in PCR reactions with pollen and leaf cDNAs. Reactions that included all three primers amplified an approximately 455 bp product in both leaves and pollen and an approximately 395 bp product in pollen only. Comparison with gene models indicated that the larger product was from RNAs transcribed from the annotated TSS and spliced using the upstream donor site, and Sanger sequencing confirmed this. However, comparison with gene models suggested that the lower-molecular weight product appearing only in pollen came from the downstream donor site (consistent with gene model AT3G06330.2) and an alternative TSS. If the annotated start site were used in conjunction with the downstream donor site, primers F1 and R would have amplified a larger product than the two we observed. These results suggested the models shown in Fig. S3B, and Sanger sequencing of the PCR products confirmed them. These results suggest two alternative TSSs were used in pollen but only the upstream site was used in leaves (Figs. S3B–S3C).

A similar result was obtained for RH11, an alternatively spliced gene encoding a DEAD/H-box RNA helicase. Inspection of the RNA-Seq alignments suggested there was at least one alternative TSS, and this alternative TSS enforced the use of an alternative donor site in the first intron (Fig. S4A). Variant-specific forward primers were used with a common reverse primer to amplify the differentially spliced regions. The variant-specific primers amplified product in both leaves and pollen, indicating that both samples used the alternative TSSs, but pollen and leaf samples each favored a different variant (Figs. S4B–S4C).

Lastly, we also found evidence for alternative TSSs in AT1G67480, which encodes a galactose oxidase/kelch repeat superfamily protein. The RNA-Seq data indicated that the annotated gene models were correct, but also suggested that other isoforms were expressed (Fig. S5A). The pollen data contained reads indicating a novel splice form, a retained-intron form, and two possible alternative TSSs. Semi-quantitative PCR amplification supported these alternative forms (data not shown). Primers designed to amplify the annotated, differentially spliced region produced amplicons with sizes that were consistent with predictions from the RNA-Seq data (Fig. S5B). Multiple variants were co-expressed in pollen and leaves, but their relative abundance was different between the sample types (Fig. S5C).

### Semi-quantitative PCR analysis of AT2G19480, a NAP1 histone chaperone, failed to show pollen specific splicing

AT2G19480 (NAP1;4) encodes a NAP1 histone chaperone (Liu et al., 2009; Gao et al., 2012) with three splice variants that differ in the three prime region. According the RNA-Seq data, an exon-skipped form was preferred in pollen and the non-skipped, retained form was preferred in leaves (Fig. S6A). PCR primers were designed to amplify all forms (F and R2 in Fig. S6A) or just the exon-included form (F and R1 in Fig. S6A). However, the PCR amplifications contradicted the RNA-Seq results in that we found evidence for the exon-skipped form only in each sample (data not shown). Thus, the PCR assessment of alternative splicing confirmed existence and expression of the exon-skipped form in both pollen and leaves but was unable to identify the exon-retained form.

### On-line data repository and RNA-Seq visualization resource

To enable readers to reproduce analyses, we assembled a git-based data and source code repository at http://www.bitbucket.org/lorainelab/pollenas. Readers familiar with the git version control program can clone the repository onto their local desktop, thus enabling them to view and retrieve older versions of the repository or use it as a basis for their own work. Tutorials on using git are available on the bitbucket.org Web site and many others. However, it is not necessary to use the git program to obtain a copy. Readers can also download the repository as a single “zip” by clicking the “download” icon on the project home page. Files can also be downloaded individually by clicking the “source” icon and then browsing the repository files and folders.

Analyses described above are organized into folders named for their contents. Typically, each folder contains a “results” sub-folder containing computer-generated results files and a “data” sub-folder containing primary data files obtained from the VisionWorks software or sequencing facility. Most analyses reported here were done using the R programming language and are documented in R “Markdown” (.Rmd) report-generation files that enable results and analysis code to be shown together in the same report file. Each “Markdown” file has a corresponding HTML report output file that was generated using the knitr R library, a tool for literate programming in R. As such, readers can either re-run the code themselves or can open the report file in a Web browser, after downloading the repository. Note that re-running analyses will also require installing some libraries that are not part of the base R distribution; this can be done using the “install packages” command within R.

We also provided RNA-Seq alignment files for visualization in Integrated Genome Browser as part of a pollen-specific IGBQuickLoad site at http://igbquickload.org/pollen. To view the data in IGB, readers first should obtain a copy of the software from the IGB web site at http://www.bioviz.org/igb. The software can be downloaded for free from the site; installers are available for Apple, Windows, and Linux computers. Once IGB is running, users can click the Arabidopsis image on the IGB home screen; this opens the Arabidopsis TAIR10 genome and loads the Arabidopsis reference gene annotations. To load RNA-Seq data shown in the figures, users can open the Pollen data source under the IGB Data Access tab. The data sets described here are named Pollen, Leaf 1, and Leaf 2. Alignments of sequences obtained from Sanger sequencing of amplicons can also be loaded. To view these data, readers can download the alignments file (named Amplicons.pslx) from the data and source code repository and open them in IGB as with any other file.

**Figure 4:**
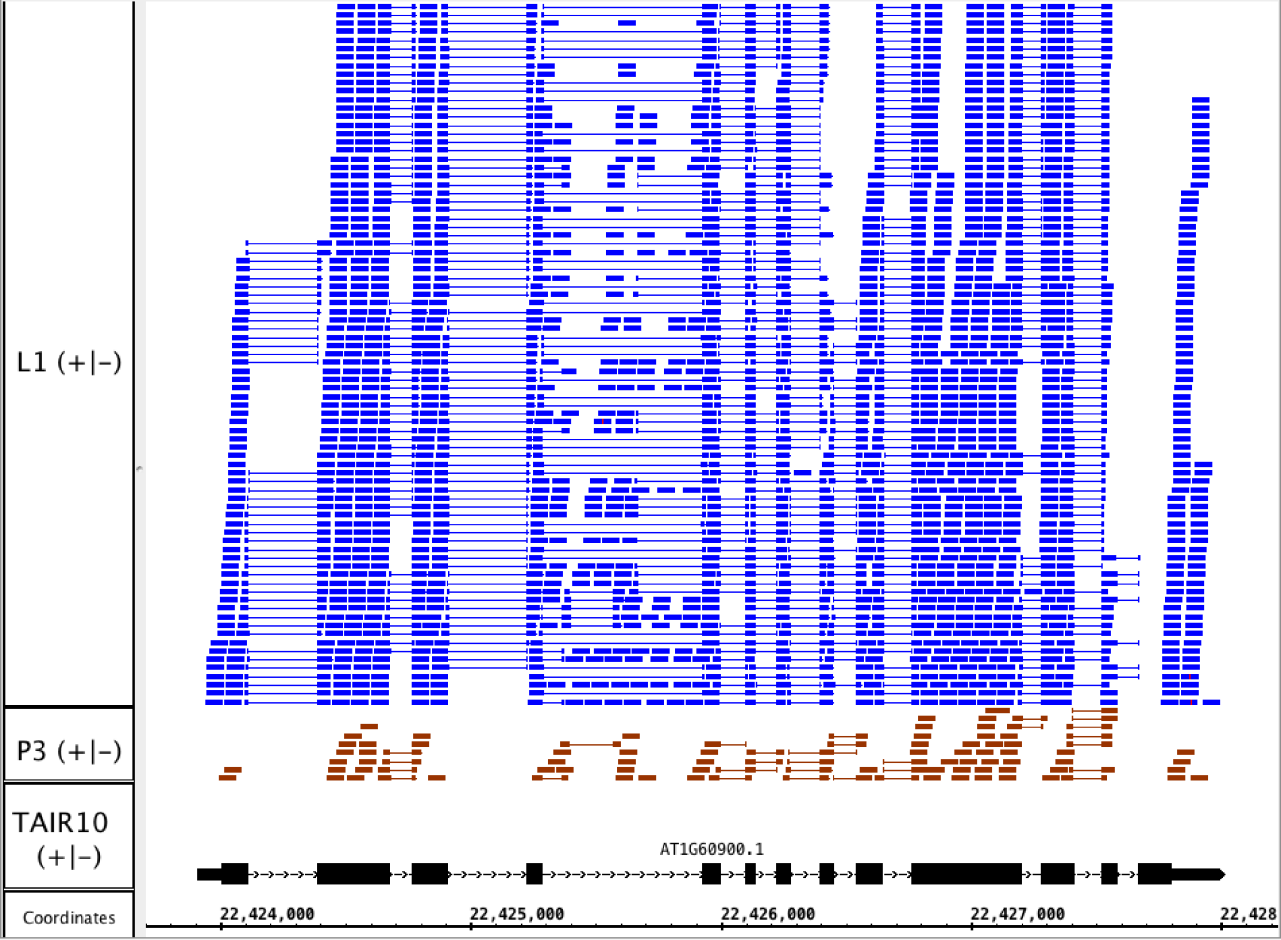
RNA-Seq read alignments indicating that the fourth intron in Arabidopsis U2AF65B is alternatively spliced. Reads from pollen sample P3 and leaf sample L1 are shown.

To illustrate the potential usefulness of this resource, we examined RNA-Seq alignments overlapping U2AF65B, one of two genes encoding U2AF65, the large subunit of the U2 snRNP accessory factor dimer, which helps to define 3′ splice sites by recruiting the U2 snRNP to the pre-mRNA. Genes that encode U2AF65 in Arabidopsis include U2AF65A (AT4G36690) and U2AF65B (AT1G60900). Previously, we showed that U2AF65A is differentially spliced in pollen as compared to leaf, but we did not report on alternative splicing of U2AF65B because there is only one model associated with this gene, and our splicing analysis pipeline detected differential splicing only in genes with multiple models. We therefore visually inspected RNA-Seq read alignments overlapping U2AF65B. Visualization of the RNA-Seq read alignments suggested that U2AF65B encodes at least one other splice variant thanks to alternative splicing of the fourth intron (Fig. 4). However, because the gene was not abundantly expressed in pollen, it was impossible to determine from RNA-Seq data alone whether the gene is differently spliced in pollen versus leaf.

## Discussion

To better characterize alternative splicing patterns in pollen, we performed semi-quantitative PCR testing of several genes that prior bioinformatic analysis of RNA-Seq data had indicated were differentially spliced between pollen and Arabidopsis leaves. For each gene, read alignments and gene models were visualized using the Integrated Genome Browser (Nicol et al., 2009). In each case, visual inspection led to deeper understanding of splicing patterns and their possible effects on protein sequence and biological function. PCR testing of independently prepared pollen and leaf samples confirmed pollen-specific splicing patterns for eight of nine genes tested. Two genes with validated pollen-specific splicing patterns encoded SR proteins (splicing regulators that influence splice site choice) and alternative splicing of the pollen-enriched forms produced isoforms with altered conserved motifs important for function. In four genes, PCR analysis showed that alternative splicing choices coincided with possible alternative transcriptional starts sites, which enforced selection of alternative donor sites at the 5′ ends of the affected genes. Table 1 summarizes the results.

**Table 1:** Summary of alternative splicing assay results. All genes except AT2G19480 were confirmed through PCR to have differential splicing patterns in pollen versus leaf sample types. Table indicates whether AS affected predicted protein, and if putative alternative transcriptional start sites (Alt. TSS) were identified.

Locus	Annotation	AS affects protein	Putative Alt. TSS
AT1G09140	At-SR30, involved in splicing	Y	N
AT5G52040	At-RS41, involved in splicing	Y	N
AT5G65685	Starch synthase	Y	N
AT4G21720	Unknown	Y	N
AT3G17120	Unknown	N	Y
AT3G06330	Zn-finger, RING-CH-type	N	Y
AT3G58510	RH11, RNA helicase	N	Y
AT1G67480	Galactose oxidase/kelch repeat	N	Y
AT2G19480	Nucleosome assembly protein	Y	N

**Notes.**

P pollen L leaves N no Y yes

Of the nine genes tested, only one gene (AT2G19480, nucleosome assembly protein) was not confirmed as differentially spliced between pollen and leaves. However, this negative result may have been due to limitations of the PCR-based assay. The differentially spliced variants differed by only 18 bases, which made distinguishing them by gel electrophoresis difficult. The difficulty of assaying this gene using the semi-quantitative PCR assay highlights the value of alternative assays with greater power to resolve variants that have similar sizes, such as assays that use gel capillary electrophoresis on an ABI sequencer to separate splice variants at single-base resolution (Simpson et al., 2008). The ability to resolve such small differences is important because genes that produce splice variants in significant amounts often have small differences between splice variants (English, Patel & Loraine, 2010). However, the method requires access to a capillary sequencer and so may not be accessible to most labs.

Of the eight genes whose splicing patterns were confirmed, PCR results strongly suggested that four genes also used alternative transcription start sites that, based on sequence composition, would naturally lead to use of downstream alternative donor sites. We noticed this after visualizing read alignments in Integrated Genome Browser, highlighting the value of visualization tools that support exploratory analysis of RNA-Seq read alignments. This potential relationship between alternative transcription and splicing was especially apparent for AT3G17120, encoding a protein of unknown function. As shown in Fig. 3, read alignments suggested that an alternative transcriptional start site was downstream of the alternative donor, making it impossible for the alternative donor to be used during splicing.

These results suggest that deeper analysis of alternative promoters using RNA-Seq data may lead to new appreciation of links between transcription initiation and splicing in Arabidopsis and other plants. However, to conduct such an analysis, new software tools are needed that can use the stacking pattern of reads to detect possible alternative TSSs, as we did visually (and manually) using the IGB software. However, this may be a difficult algorithm to implement as it relies on recognizing and interpreting patterns, tasks that human beings are well equipped to do but which may be difficult to implement in software. Moreover, it may be more practical to focus on sequence data from libraries that enrich for 5′ ends, e.g., CASE-Seq (Takahashi et al., 2012).

Of the nine genes examined here, three of the genes (At-RS41, At-SR30, RH11) had functions related to RNA processing or RNA splicing. Including At-U2AF65A, which was previously tested and had a pollen-specific splicing pattern (Loraine et al., 2013), 20% of the 30 most differentially spliced genes were functionally linked to splicing and RNA processing; others included SR protein gene At-SR34, nuclear poly(a) polymerase (nPAP), and polypyrimidine tract-binding protein (PTB2) (Data S1). This enrichment of genes involved in splicing suggests that in plants, alternative splicing provides a mechanism to fine-tune the splicing machinery to accommodate different gene expression profiles in diverse cell types. As we showed previously, the profile of gene expression in pollen was very different from that in leaves; there were many genes that were highly up regulated or expressed exclusively in pollen when compared to other samples types. Thus, correct splicing of genes expressed primarily in pollen may require a different profile of splicing regulators and effectors. Since some of these splicing effectors and regulators are expressed in multiple cell types, alternative splicing may serve to modulate their activity in diverse cellular contexts.

## Supplemental Information

10.7717/peerj.919/supp-1 Data S1 Alternative splicing events that differ the most between leaves and pollen

10.7717/peerj.919/supp-2 Supplemental Information 1 Supplementary Figures

10.7717/peerj.919/supp-3 Figure S1 Alternative splicing in AT5G65685, a putative starch synthase (A) RNA-Seq read alignments for leaf (L1) and pollen (P3) samples alongside annotated gene models with PCR primer locations of primers indicated on the coordinates track. Primer sequences were ACTGCAGCTAGGCGTTGTTT (F) and GTCGTTCCCGGTAAATTTTG (R) (B) Gel electrophoresis of PCR amplification of pollen (P1, P2, P3) and leaf (L3, L4) cDNAs and corresponding model of alternative splice variants. Estimated fragment size from gel and theoretical fragment size based on splice model found to left and right, respectively, in base pairs (bp). (C) Percent total of each observed splice variant in pollen and leaf samples quantified from gel electrophoresis in (B). Values are averages of replicate samples. Error bars indicate two standard deviations. Asterisk indicates *p*-value less than 0.05, double asterisk less than 0.01.

10.7717/peerj.919/supp-4 Figure S2 Alternative splicing in AT4G21720, a gene of unknown function (A) Junction features from RNA-Seq reads for leaf (L2, L1) and pollen (P3) samples alongside annotated gene models. Number of spliced reads supporting each junction indicated above junction. Locations of primers indicated on the coordinates track. Primer sequences were AAGGGATGTGATGCCGATAG (F) and TCCTCAGTAGGAGGCTGCAT (R) (B) Gel electrophoresis of PCR amplification of pollen (P1, P2, P3) and leaf (L3, L4) cDNAs and corresponding model of alternative splice variants. Estimated fragment size from gel and theoretical fragment size based on splice model found to left and right, respectively, in base pairs (bp). (C) Percent total of each observed splice variant in pollen and leaf samples quantified from gel electrophoresis in (B). Values are averages of replicate samples. Error bars indicate two standard deviations. Asterisk indicates *p*-value less than 0.05, double asterisk less than 0.01.

10.7717/peerj.919/supp-5 Figure S3 RNA-Seq and PCR amplification of AT3G06330 transcript variants (A) Zoomed-in view of the 5′ region of RNA-Seq read alignments from leaf (L1) and pollen (P3) alongside annotated gene models. Location of alternative transcription start site (Alt. TSS) indicated by arrow. PCR primers shown on the coordinates axis. Primer sequences were AGATTCGGCAGCTCAAGAAC (F1), TTTTCCGGGGTTGTTGATAGA (F2), and ACCCCAGCTGAAAGTTGTGT (R). (B) Gel electrophoresis of PCR amplification from pollen (P1, P2, P3) and leaf (L3, L4) cDNAs from reactions that included all three primers F1, F2, and R. Corresponding model of alternative splice variants to right of gel. Estimated fragment size from gel and theoretical fragment size based on splice model found to left and right, respectively, in base pairs (bp). (C) Percent total of each observed splice variant in pollen and leaf samples quantified from gel electrophoresis in (B). Values are averages of replicate samples. Error bars indicate two standard deviations. Asterisk indicates *p*-value less than 0.05, double asterisk less than 0.01.

10.7717/peerj.919/supp-6 Figure S4 Alternative splicing and alternative transcriptional start sites in RH11 (AT3G58510) (A) RNA-Seq read alignments covering the differentially spliced 5′ UTR region from leaf (L1) and pollen (P3) alongside annotated gene models. Location of alternative transcription start site (Alt. TSS) indicated by arrow. Locations of primers indicated on the coordinates track. Primer sequences were TCCGTTGGGTGAACGACTAC (F1), AGGGTTCCTTAATTGGTTTATTTCGT (F2), and ACCGGTTCTAACTCAGCATC (R). (B) Gel electrophoresis of PCR amplification of pollen (P1, P2, P3) and leaf (L3, L4) cDNAs from reactions that included all three primers F1, F2, and R. Estimated fragment size from gel and theoretical fragment size based on splice model found to left and right, respectively, in base pairs (bp). (C) Percent total of each observed splice variant in pollen and leaf samples quantified from gel electrophoresis in (B). Unannotated 758 bp fragment was below threshold for quantification. Values are averages of replicate samples. Error bars indicate two standard deviations. Asterisk indicates *p*-value less than 0.05, double asterisk less than 0.01.

10.7717/peerj.919/supp-7 Figure S5 Alternative splicing in AT1G67480, a putative galactose oxidase (A) RNA-Seq read alignments from leaf (L1) and pollen (P3) alongside annotated gene models. Location of two alternative transcription start sites (Alt. TSS) indicated by arrows. Locations of primers indicated on the coordinates track. Primer sequences were ACAGAACCCAAAAACCGACA (F) and ACTTTCGTGAAACCCGTCAC (R) (B) Gel electrophoresis of PCR amplification of pollen (P1, P2, P3) and leaf (L3, L4) cDNAs and corresponding model of alternative splice variants. Estimated fragment size from gel and theoretical fragment size based on splice model found to left and right, respectively, in base pairs (bp). (C) Percent total of each observed splice variant in pollen and leaf samples quantified from gel electrophoresis in (B). Values are averages of replicate samples. Error bars indicate two standard deviations. Asterisk indicates *p*-value less than 0.05, double asterisk less than 0.01.

10.7717/peerj.919/supp-8 Figure S6 Exon skipping in AT2G19480 (NAP1;4) (A) Coverage graphs for leaf (L2, L1) and pollen (P3) RNA-Seq data showing exon skipping or exon inclusion in the final intron. Values on the *y* axis are the number of reads per base pair positions indicated in the coordinates track. Locations of PCR primers are indicated on the coordinates track. Primer sequences were CAAGGACAAATGGAGCATGA (F), CTTGTGCCCAGCTGATGA (R1), and TCTCACCTGCTTGACCTTCC (R2). (B) A close-up view of the junction tracks for the alternatively spliced exon in the 3′ region. Number of spliced reads supporting each junction indicated above junction.

## Additional Information and Declarations

### Data Deposition

The following information was supplied regarding the deposition of related data: https://bitbucket.org/lorainelab/pollenas.
