# Supplementary material for: Analysis of pollen-specific alternative splicing in Arabidopsis thaliana via semi-quantitative PCR
Source: PeerJ. 2015 Apr 28;3:e919. doi: 10.7717/peerj.919 (PMC4419537; doi:10.7717/peerj.919)
Supplement: Supplemental Information 1 [file peerj-03-919-s002.pdf]

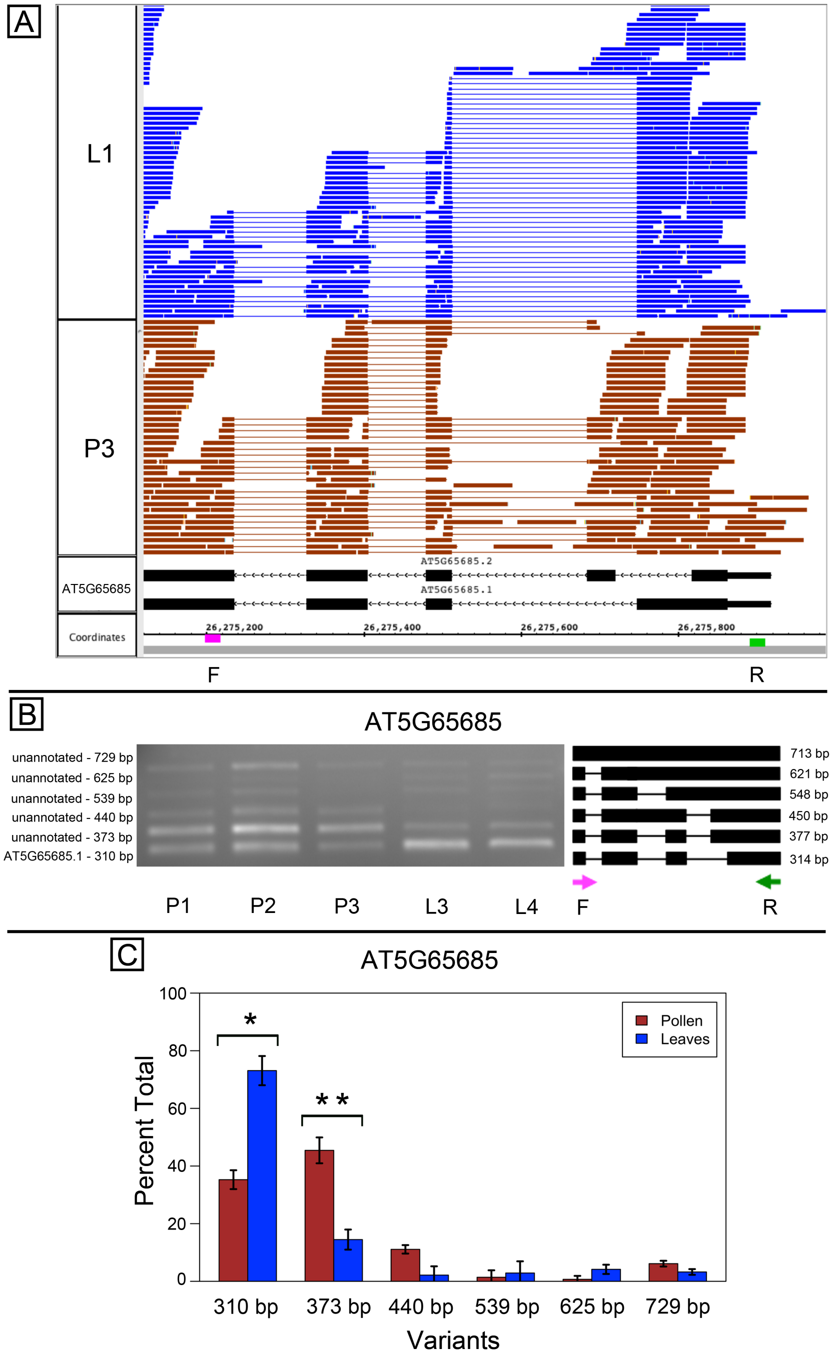

Supplemental Figure 1. Alternative splicing in AT5G65685, a putative starch synthase. (A) RNA-Seq read alignments for leaf (L1) and pollen (P3) samples alongside annotated gene models with PCR primer locations of primers indicated on the coordinates track. Primer sequences were ACTGCAGCTAGGCGTTGTTT (F) and GTCGTTCCCGGTAAATTTTG (R) (B) Gel electrophoresis of PCR amplification of pollen (P1, P2, P3) and leaf (L3, L4) cDNAs and corresponding model of alternative splice variants. Estimated fragment size from gel and theoretical fragment size based on splice model found to left and right, respectively, in base pairs (bp). (C) Percent total of each observed splice variant in pollen and leaf samples quantified from gel electrophoresis in B. Values are averages of replicate samples. Error bars indicate two standard deviations. Asterisk indicates p-value less than 0.05, double asterisk less than 0.01.

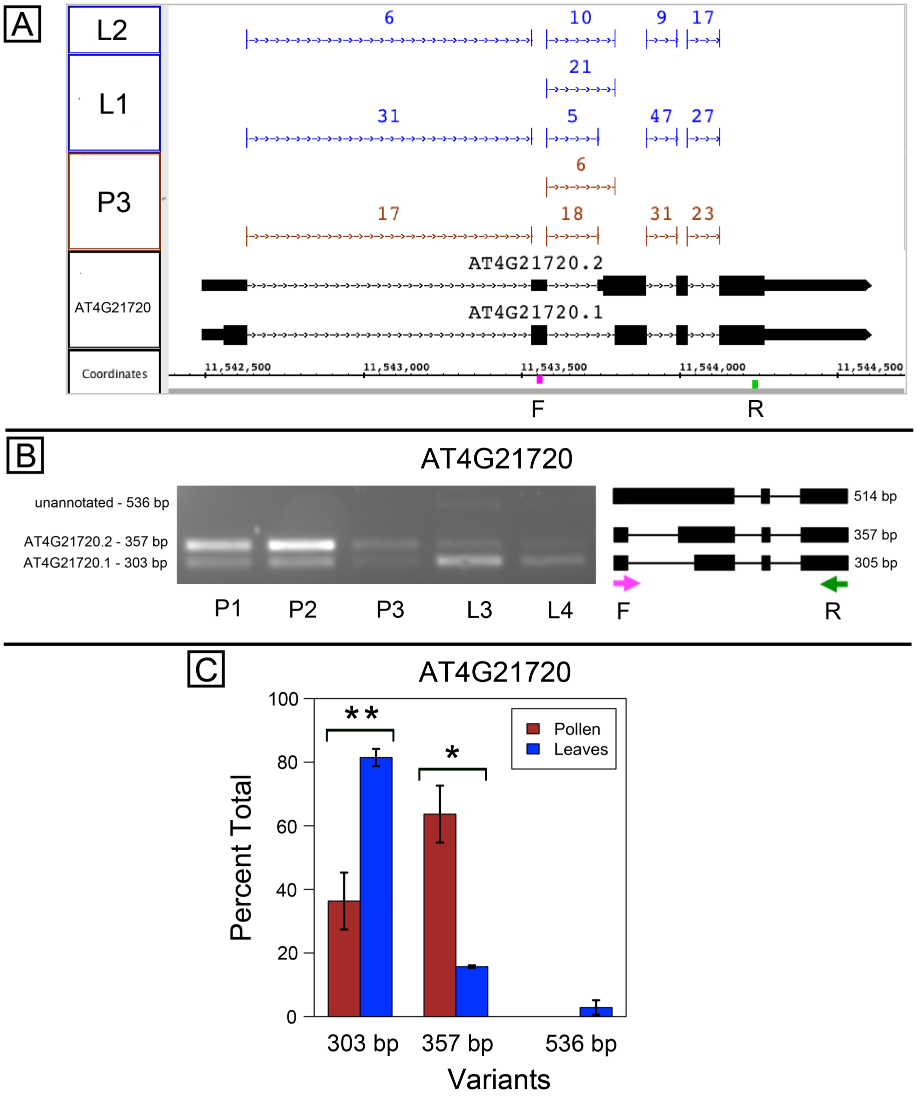

Supplemental Figure 2. Alternative splicing in AT4G21720, a gene of unknown function. (A) Junction features from RNA-Seq reads for leaf (L2, L1) and pollen (P3) samples alongside annotated gene models. Number of spliced reads supporting each junction indicated above junction. Locations of primers indicated on the coordinates track. Primer sequences were AAGGGATGTGATGCCGATAG (F) and TCCTCAGTAGGAGGCTGCAT (R) (B) Gel electrophoresis of PCR amplification of pollen (P1, P2, P3) and leaf (L3, L4) cDNAs and corresponding model of alternative splice variants. Estimated fragment size from gel and theoretical fragment size based on splice model found to left and right, respectively, in base pairs (bp). (C) Percent total of each observed splice variant in pollen and leaf samples quantified from gel electrophoresis in B. Values are averages of replicate samples. Error bars indicate two standard deviations. Asterisk indicates p-value less than 0.05, double asterisk less than 0.01.

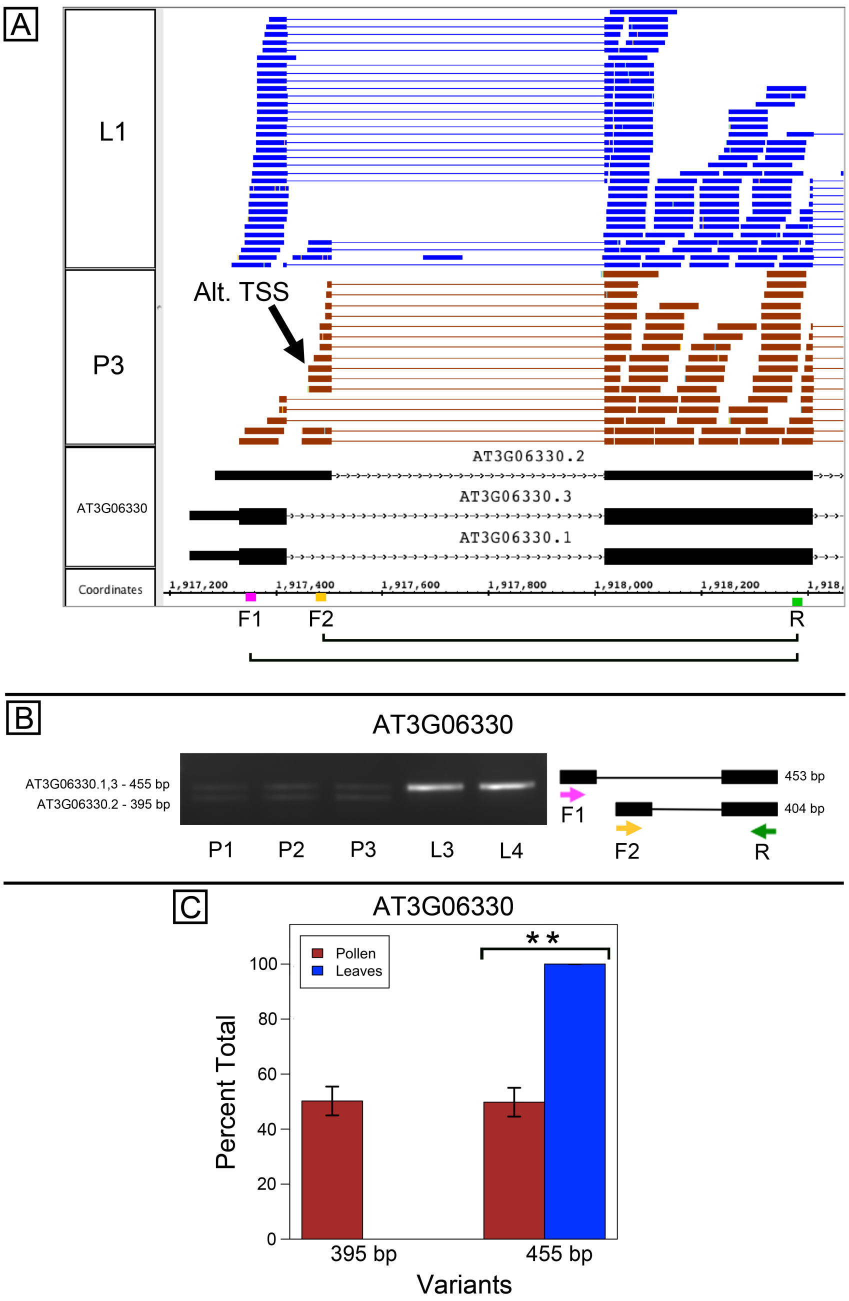

Supplemental Figure 3. RNA-Seq and PCR amplification of AT3G06330 transcript variants. (A) Zoomed-in view of the 5' region of RNA-Seq read alignments from leaf (L1) and pollen (P3) alongside annotated gene models. Location of alternative transcription start site (Alt. TSS) indicated by arrow. PCR primers shown on the coordinates axis. Primer sequences were AGATTCGGCAGCTCAAGAAC (F1), TTTTCCGGGGTTGTTGATAGA (F2), and ACCCCAGCTGAAAGTTGTGT (R). (B) Gel electrophoresis of PCR amplification from pollen (P1, P2, P3) and leaf (L3, L4) cDNAs from reactions that included all three primers F1, F2, and R. Corresponding model of alternative splice variants to right of gel. Estimated fragment size from gel and theoretical fragment size based on splice model found to left and right, respectively, in base pairs (bp). (C) Percent total of each observed splice variant in pollen and leaf samples quantified from gel electrophoresis in B. Values are averages of replicate samples. Error bars indicate two standard deviations. Asterisk indicates p-value less than 0.05, double asterisk less than 0.01.

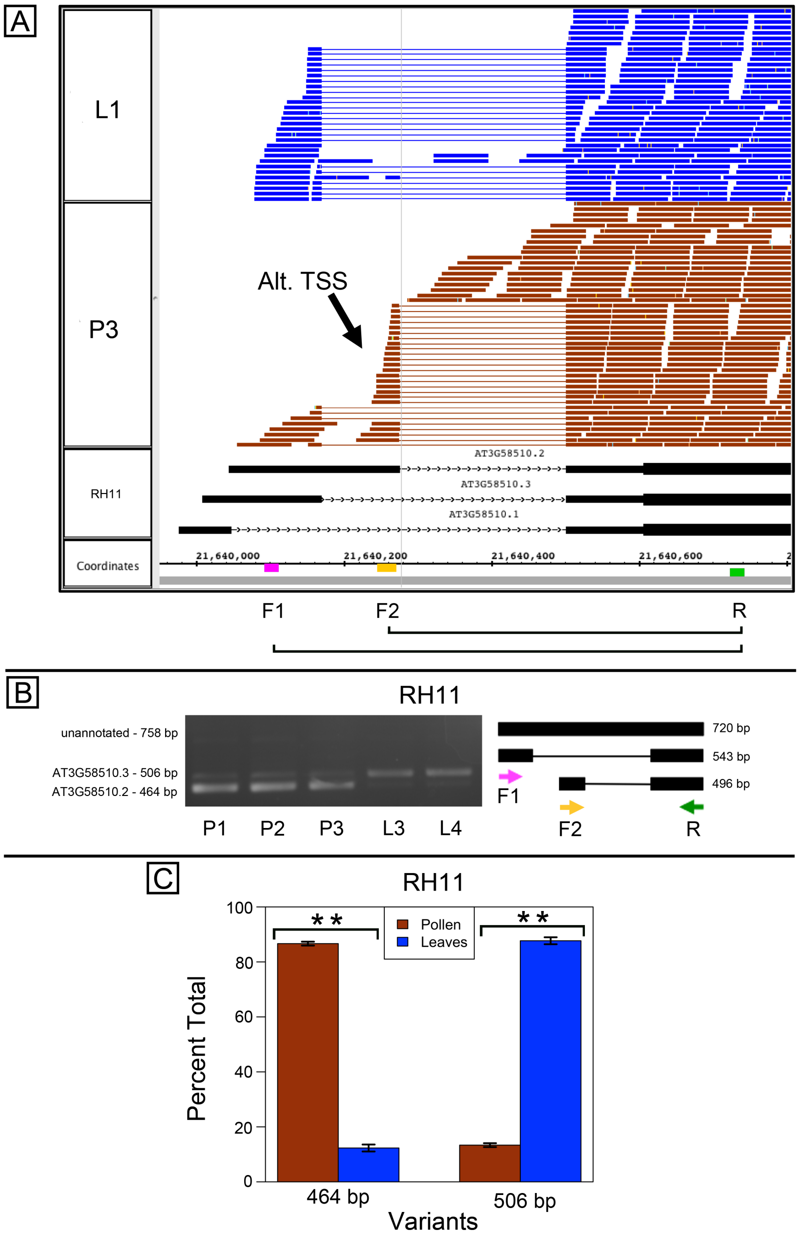

Supplemental Figure 4. Alternative splicing and alternative transcriptional start sites in RH11 (AT3G58510) (A) RNA-Seq read alignments covering the differentially spliced 5' UTR region from leaf (L1) and pollen (P3) alongside annotated gene models. Location of alternative transcription start site (Alt. TSS) indicated by arrow. Locations of primers indicated on the coordinates track. Primer sequences were TCCGTTGGGTGAACGACTAC (F1), AGGGTTCCTTAATTGGTTTATTTCGT (F2), and ACCGGTTCTAACTCAGCATC (R). (B) Gel electrophoresis of PCR amplification of pollen (P1, P2, P3) and leaf (L3, L4) cDNAs from reactions that included all three primers F1, F2, and R. Estimated fragment size from gel and theoretical fragment size based on splice model found to left and right, respectively, in base pairs (bp). (C) Percent total of each observed splice variant in pollen and leaf samples quantified from gel electrophoresis in B. Unannotated 758 bp fragment was below threshold for quantification. Values are averages of replicate samples. Error bars indicate two standard deviations. Asterisk indicates p-value less than 0.05, double asterisk less than 0.01.

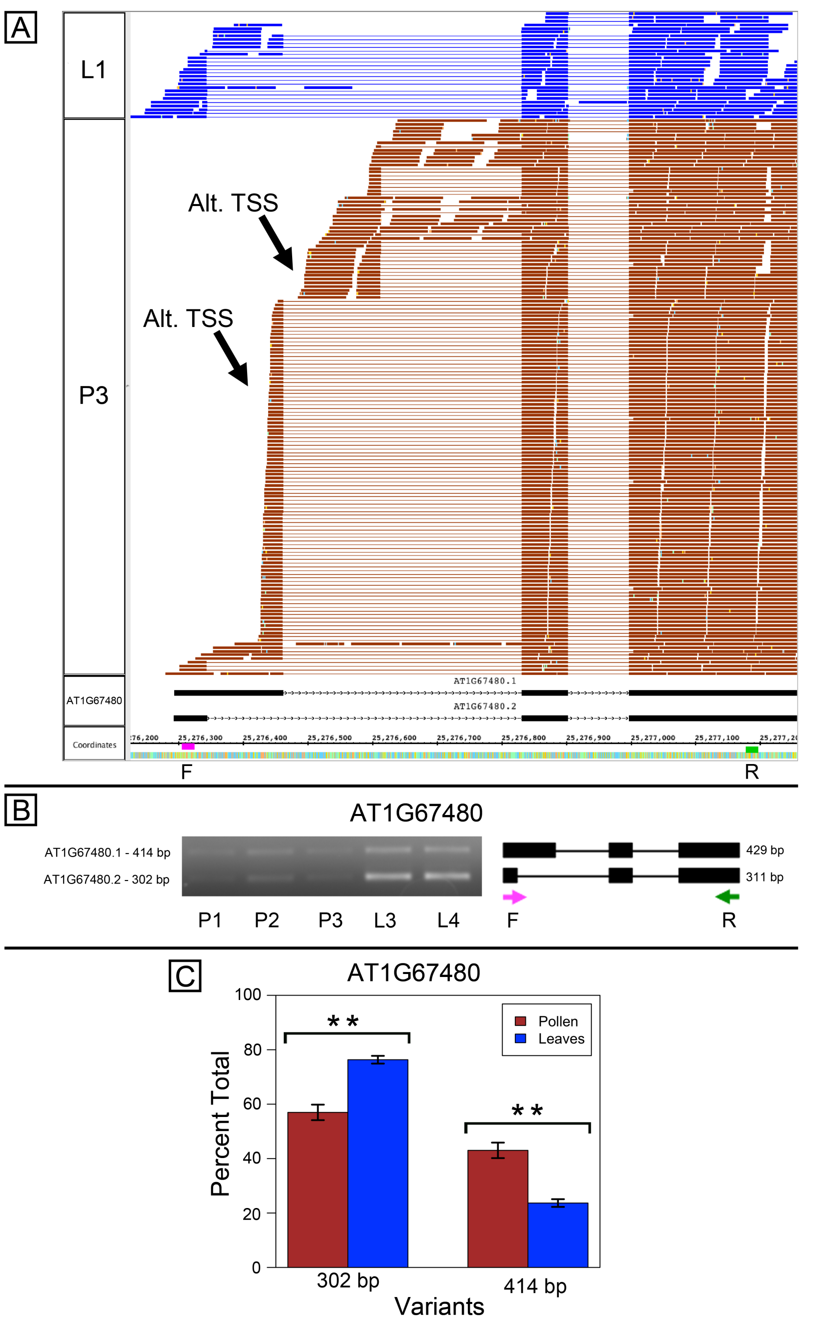

Supplemental Figure 5. Alternative splicing in AT1G67480, a putative galactose oxidase. (A) RNA-Seq read alignments from leaf (L1) and pollen (P3) alongside annotated gene models. Location of two alternative transcription start sites (Alt. TSS) indicated by arrows. Locations of primers indicated on the coordinates track. Primer sequences were ACAGAACCCAAAAACCGACA (F) and ACTTTCGTGAAACCCGTCAC (R) (B) Gel electrophoresis of PCR amplification of pollen (P1, P2, P3) and leaf (L3, L4) cDNAs and corresponding model of alternative splice variants. Estimated fragment size from gel and theoretical fragment size based on splice model found to left and right, respectively, in base pairs (bp). (C) Percent total of each observed splice variant in pollen and leaf samples quantified from gel electrophoresis in B. Values are averages of replicate samples. Error bars indicate two standard deviations. Asterisk indicates p-value less than 0.05, double asterisk less than 0.01.

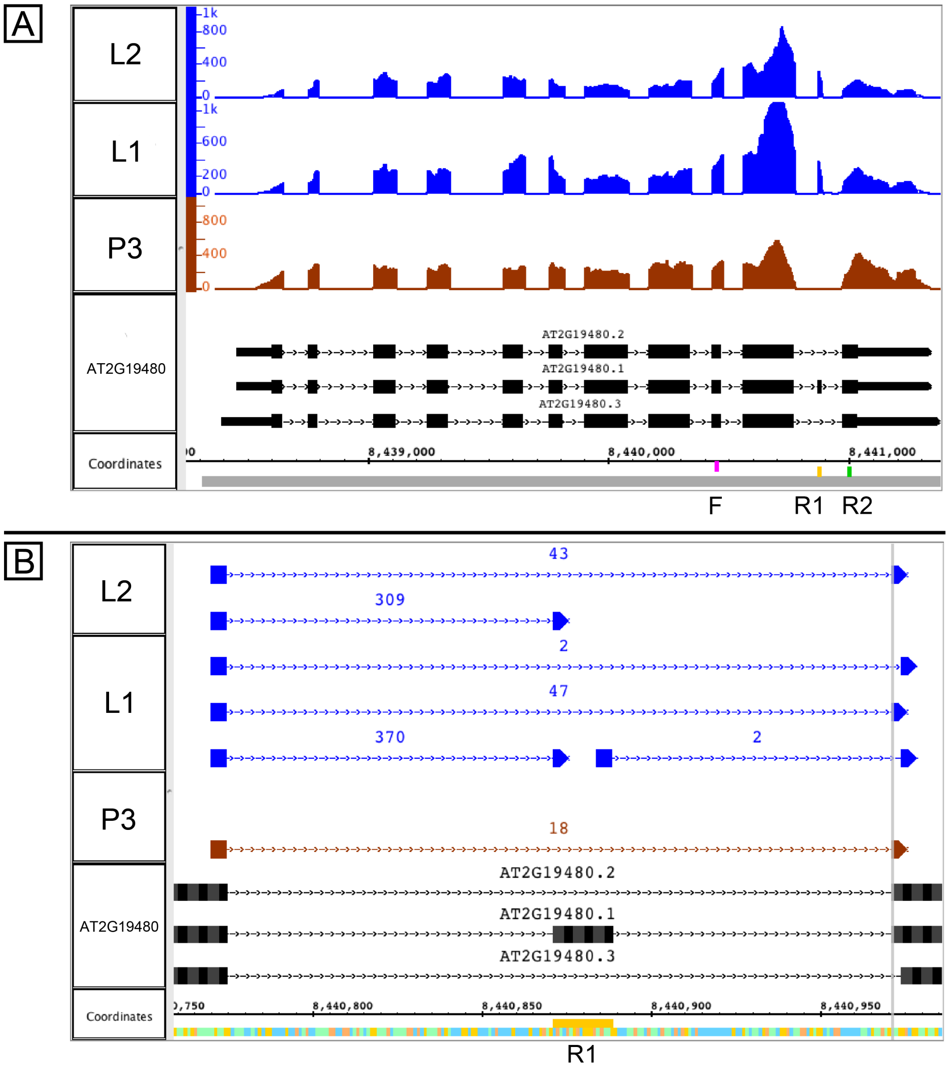

Supplemental Figure 6. Exon skipping in AT2G19480 (NAP1;4). (A) Coverage graphs for leaf (L2, L1) and pollen (P3) RNA-Seq data showing exon skipping or exon inclusion in the final intron. Values on the y axis are the number of reads per base pair positions indicated in the coordinates track. Locations of PCR primers are indicated on the coordinates track. Primer sequences were CAAGGACAAATGGAGCATGA (F), CTTGTGCCCAGCTGATGA (R1), and TCTCACCTGCTTGACCTTCC (R2). (B) A close-up view of the junction tracks for the alternatively spliced exon in the 3' region. Number of spliced reads supporting each junction indicated above junction.
